# Microwave-assisted preparation of polysubstituted imidazoles using Zingiber extract synthesized green Cr_2_O_3_ nanoparticles

**DOI:** 10.1038/s41598-022-24364-6

**Published:** 2022-11-19

**Authors:** Leila Kafi-Ahmadi, Shahin Khademinia, Ahmad Poursattar Marjani, Ehsan Nozad

**Affiliations:** 1 Department of Inorganic Chemistry, Faculty of Chemistry, Urmia University, Urmia, Iran; 2 Department of Inorganic Chemistry, Faculty of Chemistry, Semnan University, Semnan, Iran; 3 Department of Organic Chemistry, Faculty of Chemistry, Urmia University, Urmia, Iran

**Keywords:** Catalysis, Green chemistry

## Abstract

Cr_2_O_3_ nanoparticles were prepared using Zingiber officinal extract which were used as an efficient and reusable catalyst in the practical synthesis of polysubstituted imidazoles by means of a convenient reaction of aromatic aldehydes with ammonium acetate and benzil under microwave irradiation and H_2_O as solvent. The structure of the compounds was studied by IR and ^1^H-NMR spectrum. The most important benefits of this process are operational simplicity, reasonable reaction times, and excellent yield of products. The results show that the optimal conditions for the formation of imidazole derivatives are as follow: power of 400 W, reaction time of 4–9 min, H_2_O as a solvent, and 15 mmol of catalyst amount.

## Introduction

In recent years, metal and metal oxide nanomaterials have attracted significant attention in various synthesis processes^1^. Functional nanomaterials have stupendous applications in different areas such as biomedical, environment, food preservation, and health care, cosmetics, water purification, fuel cells, drug delivery and gene delivery, defense, chemical industries, space industries, ceramics, energy, sensors, single-electron transistors, textiles, agriculture, solar cells, catalysis, light emitters, fuel, and antimicrobial^2^. Among the metal oxides, Cr_2_O_3_ is more considerable due to their specific thermodynamic stability, antiferromagnetic, chemical resistance, hardness, and good catalytic reusability attributes^3^.

Chromium oxide has various crystal states such as CrO_2_ (rutile), CrO_3_, CrO_4_, Cr_2_O_3_ (corundum), Cr_2_O_5_, and Cr_5_O_12_. In this respect, Cr_2_O_3_ is known to be the most stable magnetic-dielectric oxide^4^. Cr_2_O_3_ depicts p-type and n-type semiconductor behavior^5^ that all these characteristics make Cr_2_O_3_ a suitable material for a variety of industrial applications.

Based on previous reports, numerous studies have been accomplished about applications of Cr_2_O_3_ nanoparticles (NPs) involving sensors^6^, catalysis^7^, protective coating and green pigment^8^, fuel cell^9^, solar cell^10^, piezoelectric devices^11^, photocatalysis^12^. Moreover, Cr_2_O_3_ NPs are known to be one of the significant compounds in the field of medicine and pharmacy, having anticancer, antibacterial, antileishmanial, and antioxidant specifications^5^.

Various techniques are used to synthesize Cr_2_O_3_ nanoparticles such as hydrothermal^13^, solid thermal decomposition^14^, combustion^15^, sol-gel^16^, precipitation-gelation^17^, oxidation of chromium in oxygen^18^, sonochemical^19^ mechanochemical reaction and subsequent heat treatment^20^, laser induced deposition^21^, and biological methods^22^. Besides, there are several reports introducing the synthesis of nanoparticles by green method using extracts, including CuO^23^, Cu^24^, AgCl^25^, Ag^26^, etc.

In contrast to chemical and physical methods, biological approaches are critical because of their rapid, ease in use, economic production and less generation of waste products. Different and nearly all parts of a plant such as flowers, fruit, and leaves are consisted of bio-based components like flavonoids, alkaloids, etc. The mentioned components prove the rich ingredients of the plants and exhibit their great potential to be used as a base for medical and pharmaceutical applications^27^.

A research has been accomplished on the extract of Callistemon Viminalis and examining its possible usage as a capping reagent for Cr_2_O_3_ NPs synthesis^22^. The fabrication of Cr_2_O_3_ NPs was investigated in another research through *Callostemon viminalis* extraction that were used for biological purposes^5^. In another study by Sharma and their group^28^, the extract obtained from the Cannabis Sativa leaves was used for Cr_2_O_3_ NPs preparation. Sphere-shaped Cr_2_O_3_ NPs were fabricated with Hyphaene thebaica extraction in other research^29^. The investigations in this field are vast, and in this respect, the extractions of Artemisia herba-alba leaves^30^, Melia Azedarach fruits^30^, and Nephelium Lappaceum^31^ were used for Cr_2_O_3_ synthesizing. It has been reported that the leaves of Opuntia Ficus can be a potential reducing, and capping agent for Cr_2_O_3_ preparation^32^. Rhamnus Virgata^33^, Ipomoea batatas^34^ and Tridax Procumbens^35^ are among the other reported plants that their extraction has been used for Cr_2_O_3_ NPs production.

Effect of pH, temperature, concentration of extract and reaction time on the green synthesis of nanoparticles have been investigated. For instant, changing the pH value of the reaction mixture solution changes the grain size of the synthesized sample. Synthesis of nanoparticles in the green rout requires less than 100 °C. The temperature range governs the formed nanoparticles nature^36^. Plant extract is a complex concoction of several phytochemicals, for example, phenolics, sugars, flavonoids, xanthones, and several others. In general, it is said that hydroxyl-rich phenolics act as reducing agents for metal ions, but little is discussed about the stabilizing ligands of metal nanoparticles (NPs). Thus, despite the popularity of plant extract-mediated synthesis of NPs, the phytochemical basis of the process and the exact mechanism are still unclear^37^.

Zingiber, known as Ginger as well is one of the mainly used herbals containing bioactive compounds such as phenols, paradols, curcumin, etc. Ginger is associated with the Zingiberaceae family involving about 800 species.

**Figure 1:**
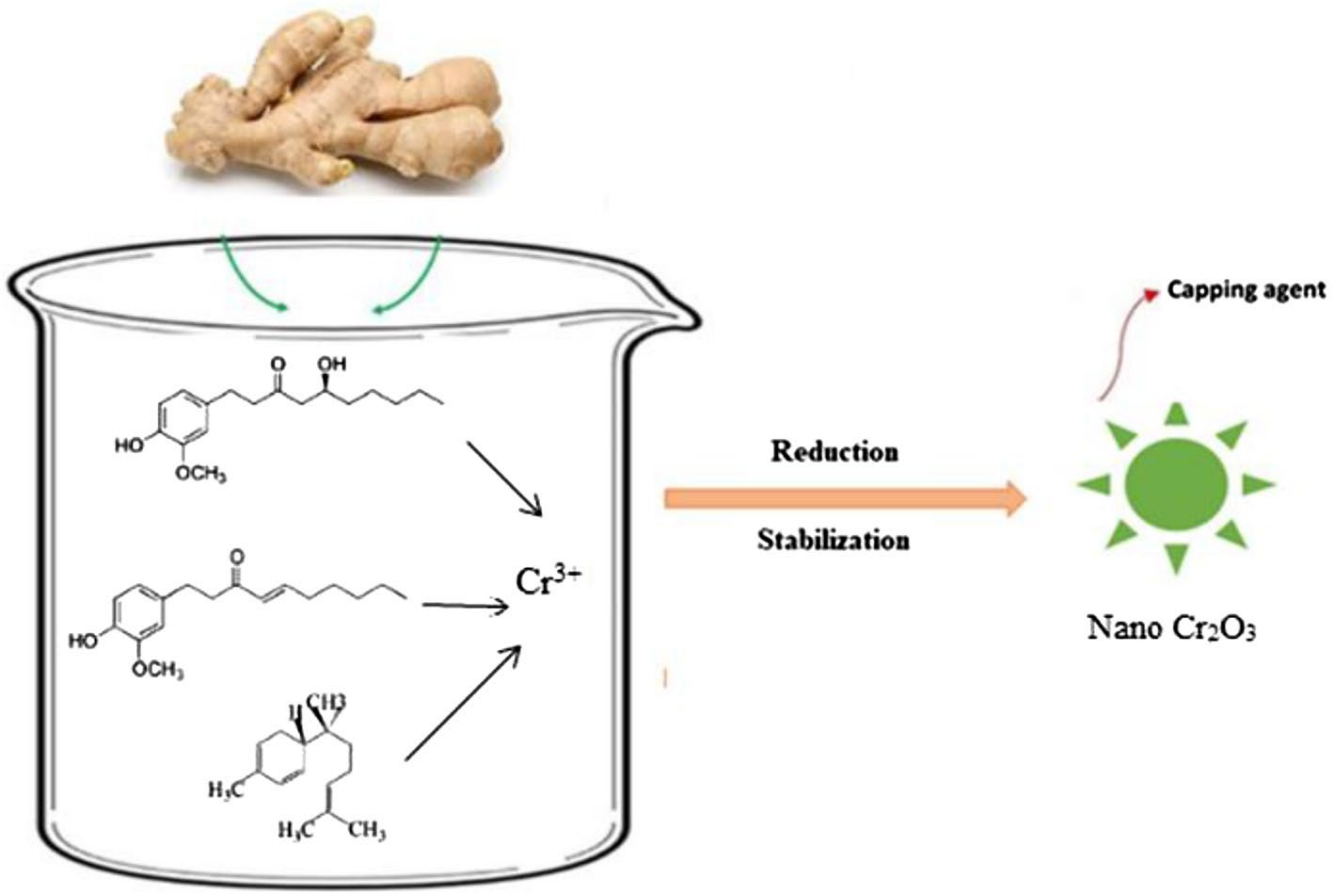
Schematic representation of the biosynthetic pathway of Cr_2_O_3_ nanoparticles using *Zingiber officinale* extract.

The ginger is used for therapeutic purposes in as much as its phytochemical’s components. This characteristic of ginger is very effective on bacterial pathogens in a wide range^38^. Zingiber extract can perform both as a reducing and stabilizing agent (Fig. 1).

**Figure 2:**
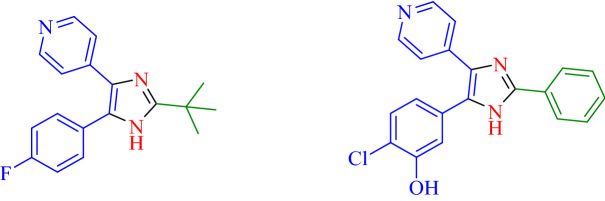
Some biological heterocyclic compounds containing imidazole moieties.

The *N*-heterocyclic compounds are considered as a group of precious compounds existing in many structures exhibiting potential features in medical materials^39,40^. In the group of numerous heterocyclic structured materials, imidazoles are very substantial within biological compounds^41^. The imidazole structural scaffolds and analogs are used as antibacterial, herbicides, fungicides, anti-inflammatory, antitumor, therapeutic, and plant growth regulators agents^42^. Moreover, this class of compound act as an inhibitor of B-Raf, p38 MAP kinase, and glucagon receptors^43^ (Fig. 2).

Such compounds are used as the base of some other structures. In this respect, imidazole synthesis obtains a high effect in the preparation of medically essential compounds. Several improved methods and procedures for the preparation of polysubstituted imidazoles have been reported, that best-reported route is a three-component, cyclo-condensation reaction between aldehyde, benzil, and NH_4_OAc in the presence of a different catalyst such as zeolite HY/silica gel^44^, ionic liquid^45,46^, iodine^47^, sodium bisulfite^48^, ZrCl_4_^49^, Yb(OTf)_3_^50^. Alternative methods with the application of microwave source energy and appropriate catalyst through using 1,2-diketone and aldehyde for imidazole synthesis have been proposed such as MW/Silica-gel^51^, glyoxylic acid^52^, InCl_3_.3H_2_O^53^.

Using the microwave energy source within the synthesis of different compounds is an environmental friendly technique. The energy of microwaves is high; therefore, short times are needed for the accomplishment of the reactions, hence, having great superiority from the time point of view. In this respect, microwave technology has an ascending usage in synthesizing various compounds^54^.

**Figure 3:**
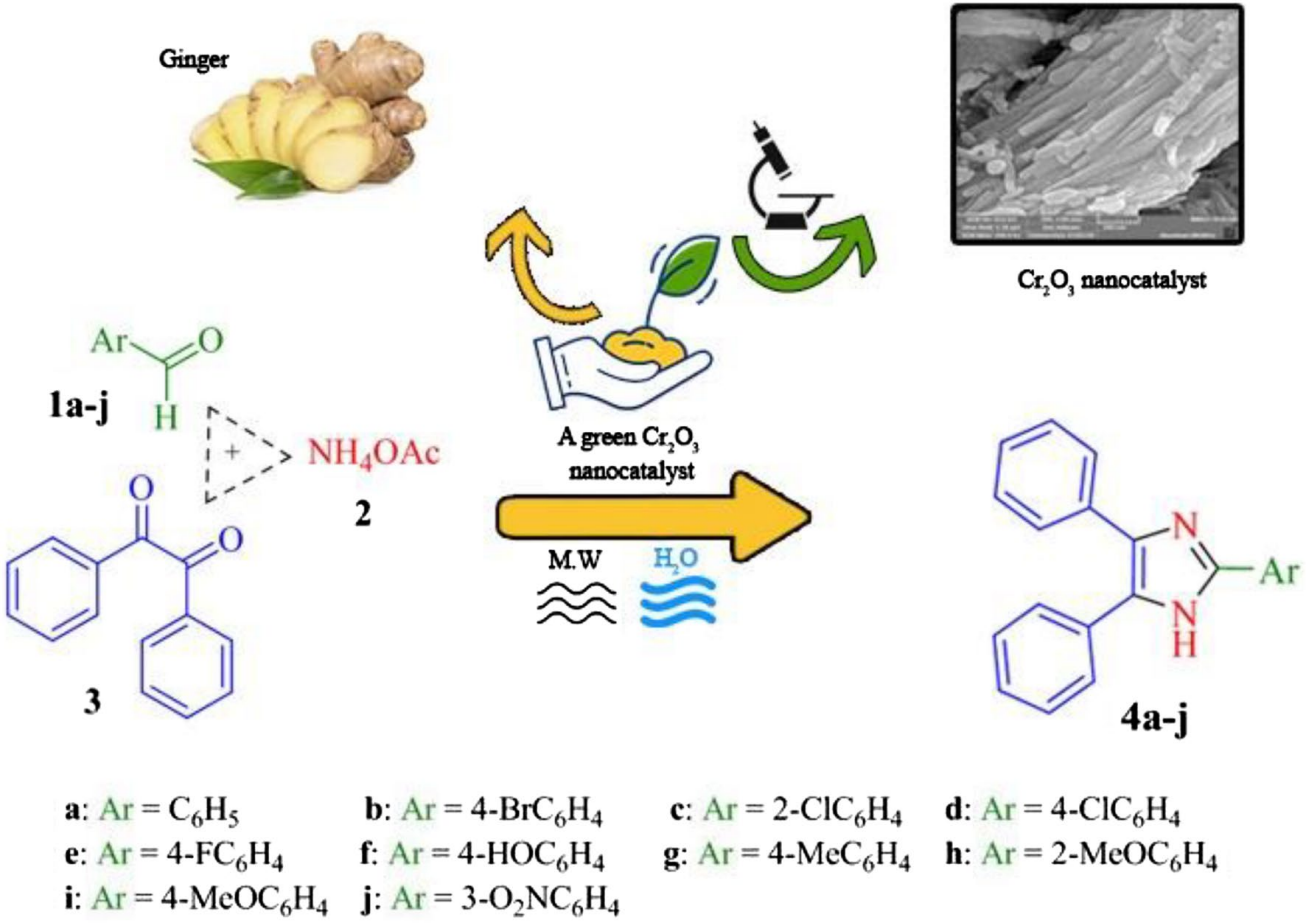
Synthesis of imidazole derivatives **4a–j** with Cr_2_O_3_ as catalyst under microwave irradiation.

In continuation of our investigation towards designing novel catalysts in the synthesis of heterocyclic compounds^55–60^, we synthesized Cr_2_O_3_ nanoparticles using Zingiber officinal extract, and used it as a Lewis acid catalyst for the preparation of polysubstituted imidazoles (Fig. 3). To the best of the authors’ knowledge, this study is the first investigation in this manner. Besides, the significance of the present study is the green and facile synthesis of the Cr_2_O_3_ nanomaterial, and application of the synthesized compound as efficient catalyst for the preparation of imidazole derivatives **4a–j**.

## Experimental

### Materials and instruments

The chemicals were obtained from Merck company, and no excess purification was carried out. Zingiber was purchased from local market, Urmia, West Azerbaijan, Iran and the collection of plants materials used in current study complied with institutional, national or international guidelines. X-ray Powder Diffraction (XRPD) pattern was recorded by the X-ray diffractometer (D5000 Siemens AG, Germany) using CuKα radiation to make phase identification. The FESEM image was taken on a Hitachi model S-4160 for morphology study. FT-IR spectra were obtained with FT-IR spectrometer (Bruker, Germany). Thin-layer chromatography using petroleum ether/ethyl acetate (9:1) mixture was used to evaluate the purity of the products. ^1^H-NMR spectra of compounds were run on a Bruker Avance DRX-400 spectrometer using tetramethylsilane as an internal standard and dimethyl sulfoxide-*d*_*6*_ as solvent. Microwave-assisted procedures were performed in the Milestone Microwave Oven.

### Preparation of plant extract

**Figure 4:**
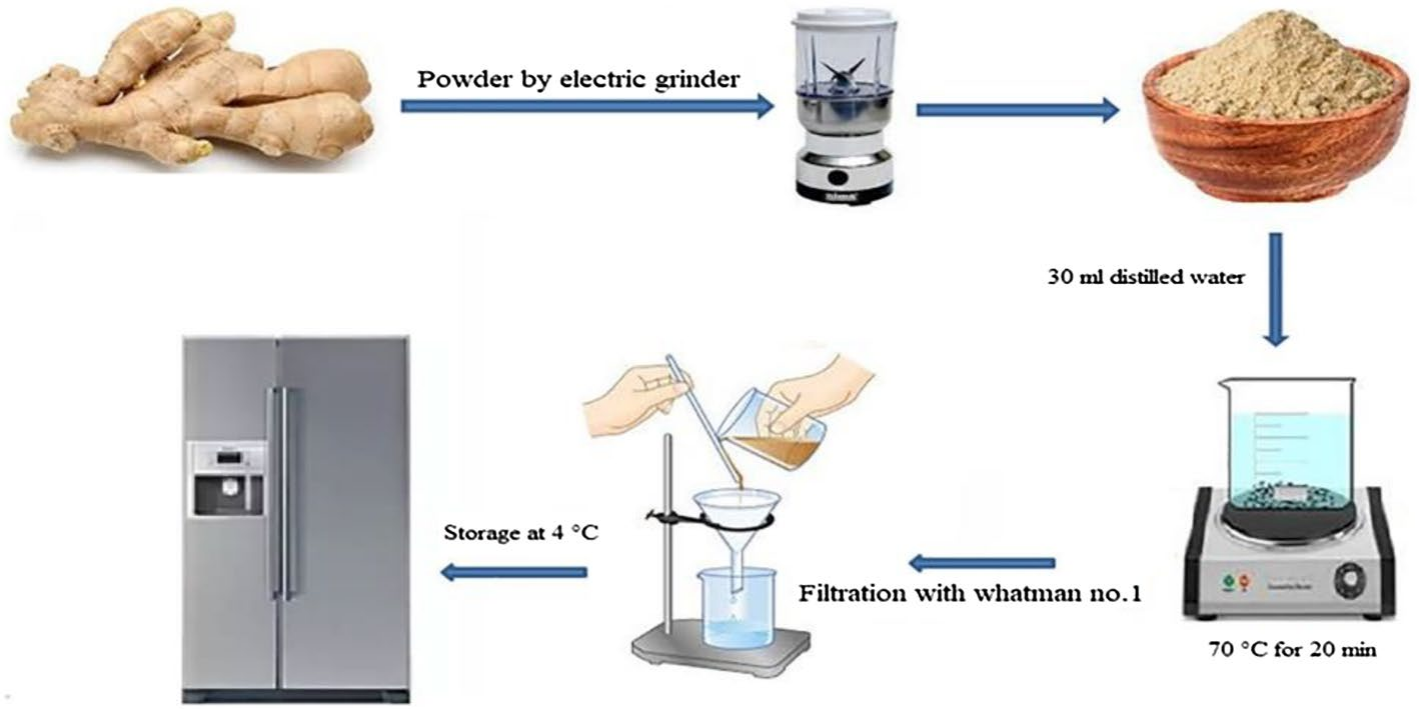
The synthesis scheme of extraction of ginger.

The purchased dried root of Zingiber was ground and a fine powder was obtained. Then, 300 mg of the prepared powder was poured into 30 mL of distilled water. The mixture was stirred at 70 °C for 20 min. Finally, the extraction was cooled to room temperature and filtered out. The product was kept at decreased temperature (4 °C) for subsequent use (Fig. 4).

### Green synthesis of Cr_2_O_3_ nanoparticles

**Figure 5:**
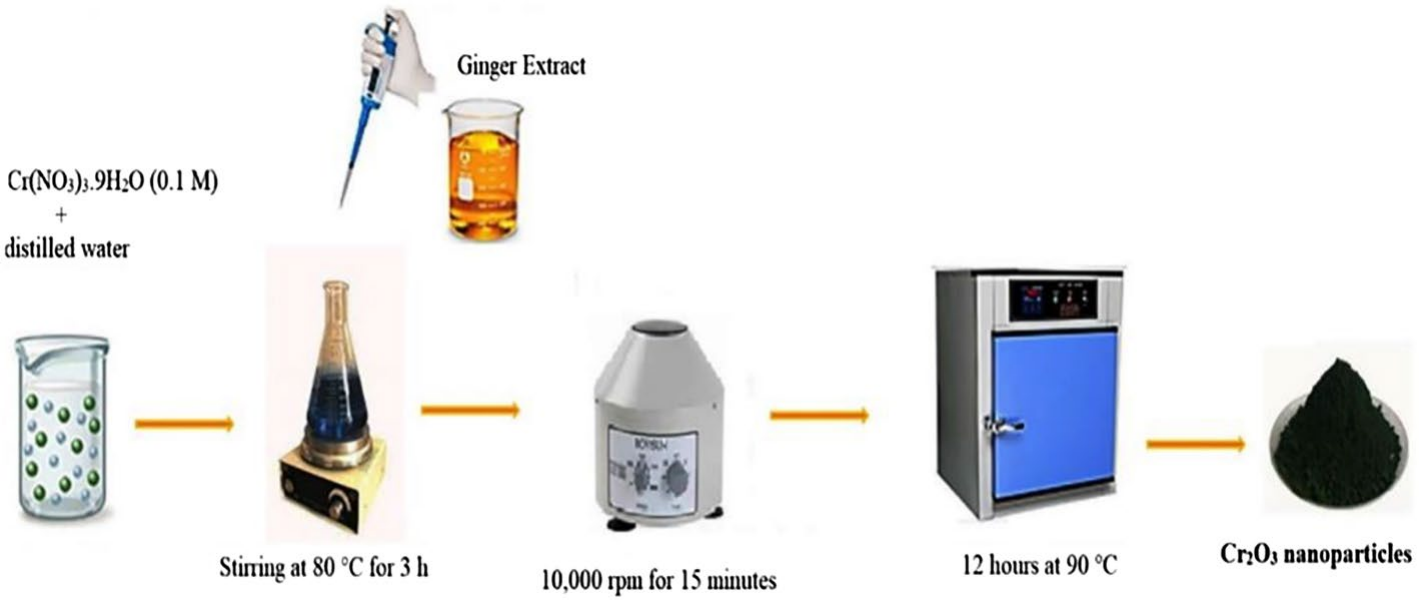
The stepwise synthesis pathway of Cr_2_O_3_ nanoparticles.

Ginger aqueous extract (3 mL) was added to Cr(NO_3_)_3_·9H_2_O (0.1 M) under continuous stirring. Sodium hydroxide 2 M solution was used to adjust the pH on 12 at 80 °C. The formed precipitate was centrifuged for 15 min, then rinsed with distilled water, and dried at 90 °C for 12 h in an oven (Fig. 5).

### General procedure for the synthesis of imidazole derivatives 4a–j

A mixture of aromatic aldehydes (**1a–j**, 1 mmol), ammonium acetate (**2**, 3 mmol), and benzil (**3**, 1 mmol) in water (2 mL), and Cr_2_O_3_ nanoparticles (15 mmol) were prepared. The obtained mixture was kept under agitation and microwave (400 W) was used to treat the mixture with irradiation for an appropriate time (Table 5, reaction time in the range of 4–9 min). TLC was used to investigate the reaction progress (ethyl acetate/petroleum ether; 1:9 as eluent). Next, the obtained mixture temperature was decreased to room temperature by adding it to an ice containing beaker. Afterward, the achieved product was filtered out under reduced pressure, following by rinsing with water for several times and drying. Finally, recrystallization was done using ethanol in order to obtain a highly pure products **4a–j** (89–98% yield).

## Results and discussions

### Powder X-ray diffractometry analysis

**Figure 6:**
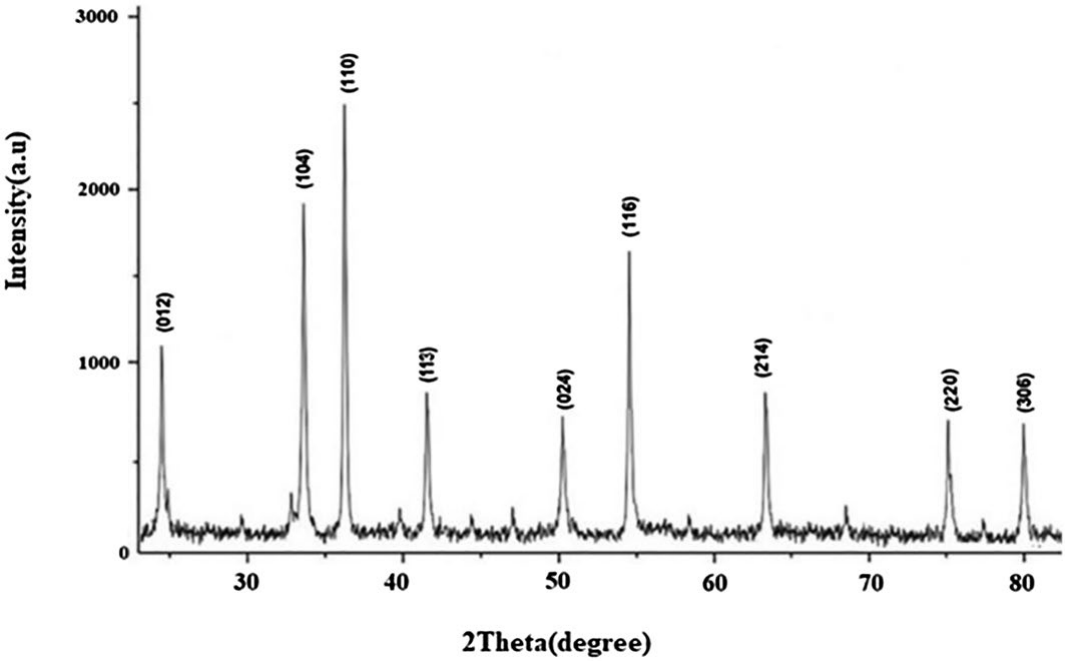
XRD analysis of green synthesized Cr_2_O_3_ nanoparticles.

XRD pattern of the prepared catalyst is shown in Fig. 6, and nine different Bragg’s diffraction peaks can be observed associated with crystal planes of (012), (104), (110), (113), (024), (116), (214), (220), and (306) at 2θ = 24.3°, 33.7°, 36.3°, 41.4°, 50.1°, 54.8°, 63.5°, 76.7°, and 79.0° respectively. The obtained pattern for Cr_2_O_3_ nanoparticles is in agreement with Joint Committee on Powder Diffraction Standards (JCPDS) 38–1479^61^. No peak related to any impurity was seen that confirm the high purity of the particles.

In addition, the mean crystallite size of the Cr_2_O_3_ sample was evaluated using the Scherrer formula as follows:

$${\text{D }} = {\text{ K}}\lambda /\left( {\beta {\text{ cos }}\theta } \right)$$

where K (0.9), λ (1.54056 Å), β, and θ are Scherer constant, X-ray radiation wavelength, full peak width at half maximum, and Bragg diffraction angle, respectively. In this respect, Cr_2_O_3_ crystallite size, in average was calculated at about 14 nm.

### FT-IR analysis

**Figure 7:**
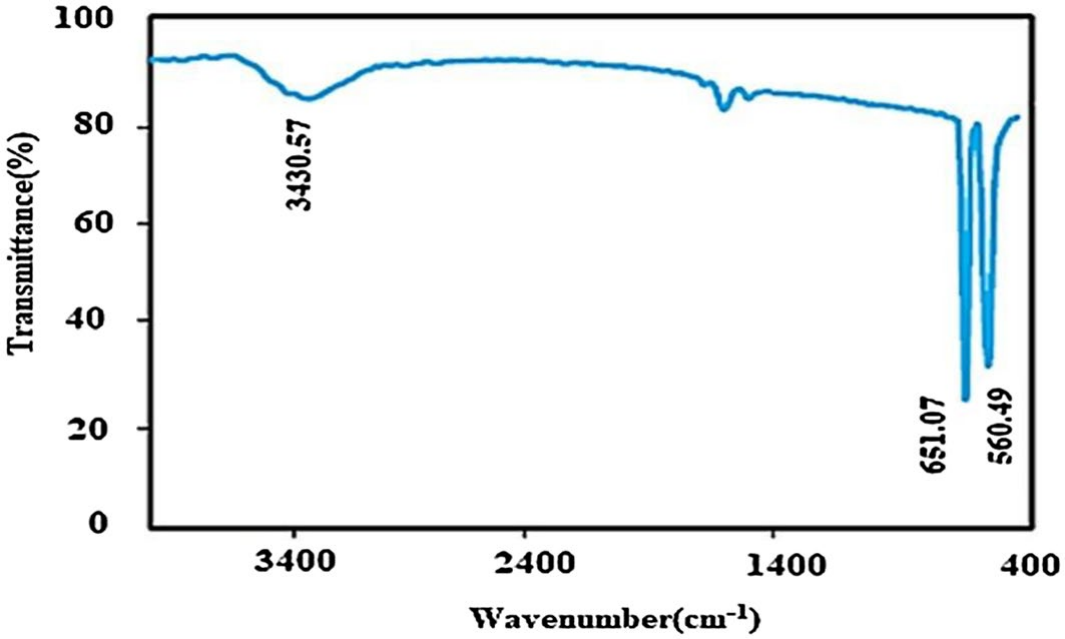
FT-IR spectrum of the green synthesized Cr_2_O_3_.

The FT-IR spectrum of Cr_2_O_3_ nanoparticles is illustrated in Fig. 7. The peaks below 1000 cm^−1^ may be due to the inter-atomic vibrations and in this study, may be associated with the Cr–O bands. This phenomenon can be seen in the spectrum of the metal oxide frequently. The high intensity of the peaks of Cr_2_O_3_ bands indicates the good crystalline nature of the material. Two sharp peaks at 651 cm^−1^ and 560 cm^−1^ could be related to Cr–O stretching modes are clear evidence of the attendance of the crystalline Cr_2_O_3_^62^. The broadband around 3400 cm^−1^ can be due to the hydroxyl groups of water.

### Morphology analysis

**Figure 8:**
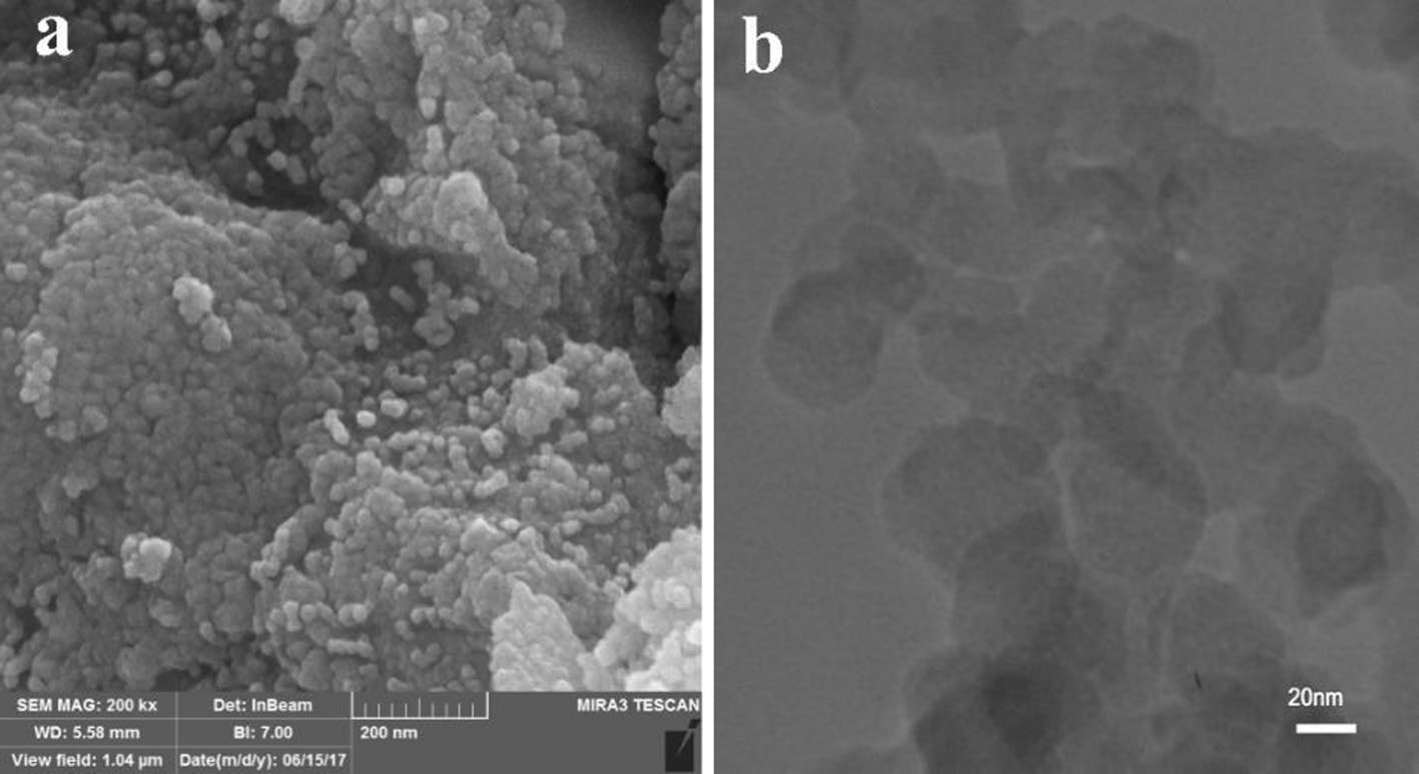
FESEM (**a**) and TEM (**b**) image of synthesized Cr_2_O_3_.

Figure 8, shows the FESEM image of the as-synthesized nanomaterial. It can be seen that the main morphology of the material is a mixture of rod and particle. TEM image exhibits that the diameter size of the as-prepared sample is 30–40 nm.

### Magnetic property

**Figure 9:**
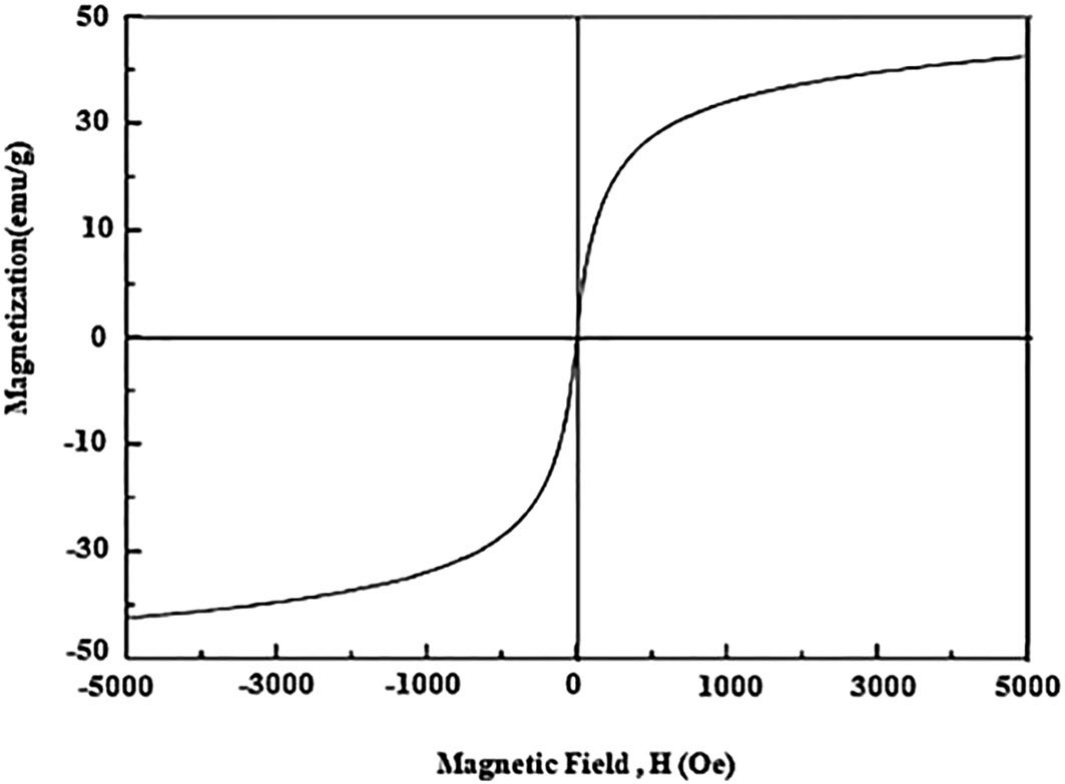
VSM curves of the as-synthesized nanomaterial.

Figure 9, shows the hysteresis loop for a sample at room temperature. Accordingly, the synthesized particle possesses a soft magnetic nature. The value of magnetization saturation (M_s_) is about 42 emu/g. Remnant magnetization M_r_ is the magnetization strength of the materials remaining after removing the external magnetic field or descending to a zero level. M_rs_ as the square of magnitudes is achieved using (M_rs_ = M_r_/M_s_) formula. Magnetic parameters are summarized in Table 1. The particle with homogenous distribution and magnetization with no inter-grain interactions will give a M_rs_ below 0.5. This value can be interpreted through multiple domains of the structure formed due to the exchange coupling among adjoining grains. In this study, the M_rs_ is 0.19 affirming that the sample has no preferred direction in magnetization. Moreover, a normal (S-shaped) narrow hysteresis loop was observed as well. The narrow loop is an indication of a low coercivity. Therefore, the prepared sample can be easily demagnetized.

**Table 1:** Magnetic parameters of the sample.

Sample	M_r_ (emu/g)	M_s_ (emu/g)	M_rs_ = M_r_/M_s_	H_c_ (Oe)
Cr_2_O_3_	4.9	42.3	0.115	50.7

## Evaluation of the catalytic activity of the as-prepared Cr_2_O_3_ nanoparticles for the synthesis of imidazoles 4a–j

The synthesized catalyst was evaluated, and their efficiency in the imidazoles preparation was studied. The one-pot, three-component reaction of benzaldehyde (**1a**), ammonia source (**2**, AcONH_4_), and benzil (**3**), was chosen as a trial reaction. Some prerequisite conditions were assessed by initial experiments regarding the optimum conditions.

Different aryl aldehydes **1a–j** were used to evaluate the reaction process. Manifestly, in the reactions with no nanocatalyst usage, no considerable progress was observed. Hence, in order to investigate the as-prepared nanocatalyst effect in the present procedure, different amounts of catalyst ranging from 5 to 25 mmol was applied (Table 2). Interestingly, excellent yield was observed using 15 mmol of nanocatalyst (Table 2, entry 4) in water as a green solvent.

**Table 2:** Effect of Cr_2_O_3_ nanoparticles as nanocatalyst on the synthesis yields of compound **4a**.

Entry	Catalyst (mmol)	Time (min)	Yield (%)
1	–	6	**–**
2	5	6	35
3	10	6	51
4	**15**	**6**	**97**
5	20	6	97
6	25	6	97

Reaction conditions: Benzaldehyde (**1a**, 1 mmol), AcONH_4_ (**2**, 3 mmol), benzil (**3**, 1 mmol), and catalyst in H_2_O (2 mL) under microwave irradiation (400 W).

Significant values are in bold.

Moreover, increase in the amount of catalyst had no significant effect on the outcome (Table 2, entries 5 and 6). Also, the lower quantities of the nanocatalyst afford moderate yield of the product at a longer reaction time (Table 2, entries 2 and 3).

Microwave effect with power in the range of 200–500 W was exanimated (Table 3). According to the results, 400 W was chosen as the optimized power for synthesizing substituted imidazole derivatives.

**Table 3:** Effect of microwave power on the synthesis of trial reaction.

Entry	Catalyst (mmol)	Microwave power (W)	Time (min)	Yield^a^ (%)
1	15	200	6	65
2	15	300	6	80
3	**15**	**400**	**6**	**97**
4	15	500	6	97

Reaction conditions: **1a** (1 mmol), **2** (3 mmol), **3** (1 mmol), and catalyst (15 mmol) in water (2 mL).

Significant values are in bold.

^a^Isolated yield.

In addition, the solvent effect was assessed, and the results are shown in Table 4. No progress in the reaction was observed without a solvent, even after a considerable time. This finding affirms the requirement for an appropriate solvent.

**Table 4:** Optimization of reaction conditions for the synthesis of compound **4a**.

Entry	Solvent	Yield (%)
1	**–**	**–**
2	Et_2_O	**–**
3	CHCl_3_	**–**
4	DMSO	14
5	THF	17
6	DMF	33
7	CH_3_CN	28
8	**H**_**2**_**O**	**97**
9	EtOH	89
10	H_2_O/EtOH (1:1)	90
11	H_2_O/EtOH (2:1)	91

The reaction of **1a** (1 mmol), **2** (3 mmol), **3** (1 mmol), catalyst (15 mmol) and solvent (2 mL) under microwave irradiation was carried out.

Significant values are in bold.

According to the results, no considerable reaction progress was observed while using nonpolar solvents such as Et_2_O. However, by using polar aprotic solvents such as DMF, low yields were achieved. Nonetheless, polar protic solvents like water, and ethanol had a better effect and the yields of 97% and 89% were obtained for H_2_O and EtOH respectively.

Encouraged by this success, using the obtained optimum reaction parameters, the scope and efficiency of this approach were demonstrated for the synthesis of polysubstituted imidazoles **4a–j** and the results are outlined in Table 5. As can be seen, the extension of substrate scope, the different aryl aldehyde containing the various functional groups on the benzene ring such as halogens, hydroxyl, methyl, methoxy, and nitro was examined with ammonium acetate and benzil under the optimized conditions for imidazoles synthesis. However, aryl aldehyde with electron-withdrawing groups, like nitro, require more reaction time to form the product **4j** (89%).

**Table 5:** Cr_2_O_3_ catalyzed the synthesis of imidazole derivatives **4a–j**.

Entry	Aromatic aldehyde	Product	Time (min)	Yield^a^ (%)	M.p.^b^ (°C)	
					Found	Refs.
1			6	97	269–271	270–272^63^
2			5	94	249–251	250–252^64^
3			6	92	197–199	198–200^65^
4			5	93	260–263	260–265^63^
5			6	94	238–240	239–241^66^
6			4	97	234–236	235–237^63^
7			4	96	230–233	232–234^64^
8			5	96	207–209	208–210^65^
9			4	98	219–221	220–223^67^
10			9	89	300–303	302–304^65^

^a^Isolated yields.

^b^The measured melting points comparison with those in literature confirmed the products.

## Recycling of Cr_2_O_3_ nanoparticles as a catalyst under microwave irradiation

**Figure 10:**
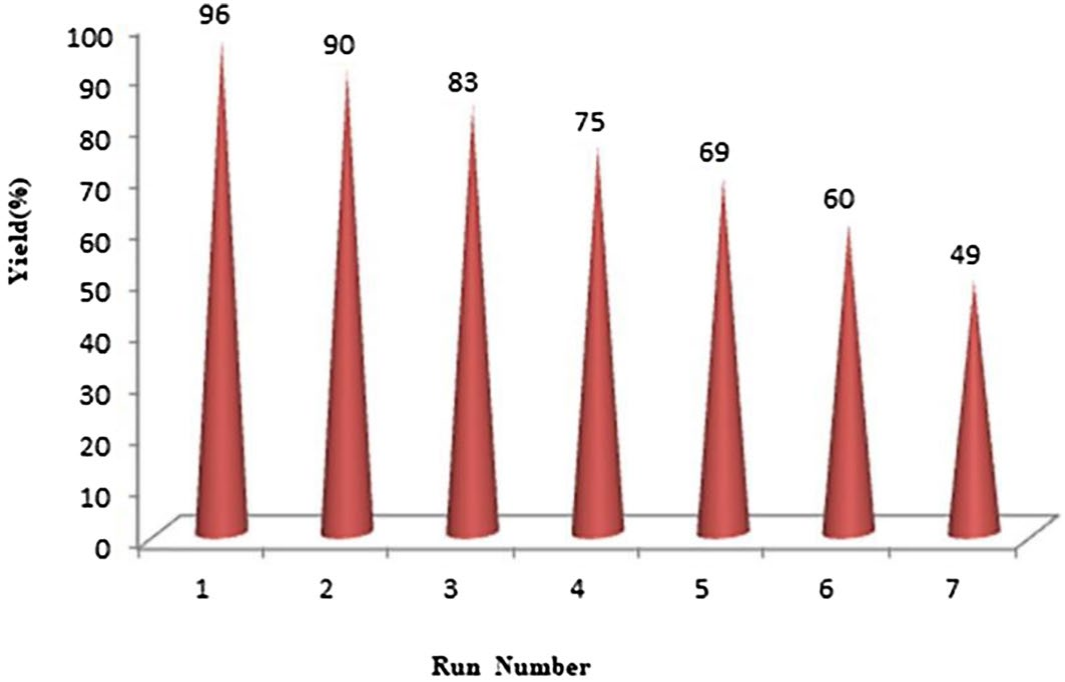
Reusability of Cr_2_O_3_ in the synthesis of compound **4a**.

The catalytic performance of Cr_2_O_3_ after multiple cycles of usage was investigated. It has been proved that the prepared nanocatalyst can be used even after 6 runs with no considerable decrease in its efficiency (Fig. 10).

To show the advantages of the current work, we compared the results with literature. As shown in Table 6, Cr_2_O_3_ is the most efficient catalyst and gives excellent product yields in reduced reaction times. In addition, the merit of Cr_2_O_3_ is its recyclability and easy work-up.

**Table 6:** Comparison of various heterogeneous catalysts in the formation of compound **4a**.

Entry	Catalyst	Time (min)	Yield (%)
1	Schiff base nickel complex (Ni–C)	20	90^68^
2	ZrO_2_–Al_2_O_3_	20	99^69^
3	Rochelle salt	10	93^70^
4	Fe_3_O_4_	20	85^71^
5	TiCl_4_–SiO_2_	10	93^72^
6	CuCl_2_·2H_2_O	13	87^54^
7	Triflate	30	96^73^
8	BTPPC	10	92^74^
9	MgAl_2_O_4_	14	93^75^
10	Cr_2_O_3_	6	97 (this work)

**Figure 11:**
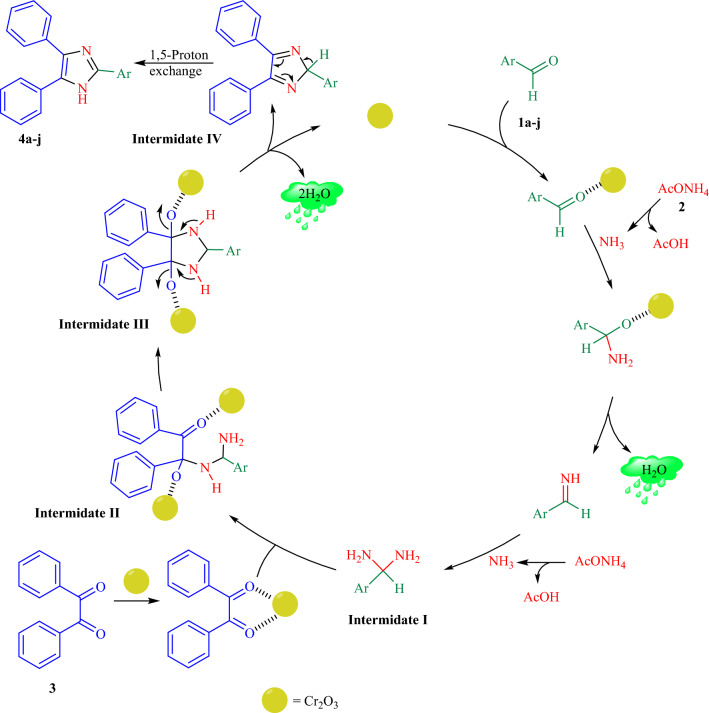
Plausible reaction mechanism for the synthesis of imidazole derivatives.

Figure 11, presents a suggested synthesis mechanism for the imidazoles. In the first step, the catalyst increased the electrophilicity of the aromatic aldehydes carbonyl groups **1a–j**. Then, the ammonia's nitrogen (**2**, obtained from ammonium acetate) intermolecular nucleophilic attack to the activated center of the carbonyl group generated diamine intermediate **I**. Next, intermediate **II **is produced by nucleophilic attack of the intermediate **I **nitrogen to the carbonyl groups of benzil (**3**). Afterwards, the typical intramolecular condensation of the intermediate **II** followed by a heterocyclization, afforded the intermediate **III**, while the removal of two water molecules occurs and the conjugate intermediate **IV** is obtained. Finally, the aromatization of intermediate **IV** takes place leads to the corresponding five membered heterocyclic compounds as the desired imidazoles **4a–j** under 1,5-proton exchange.

## Conclusion

In summary, an easy, cost-effective, and eco-friendly biological successful technique was used for synthesizing of Cr_2_O_3_ nanostructures using Cr(NO_3_)_3_.9H_2_O as a precursor, and Zingiber officinal extract as a stabilizing and reducing agent. The green synthesized Cr_2_O_3_ nanoparticles was characterized using SEM, XRD, TEM, FT-IR, and VSM analyses. The mean crystallite size was 14 nm, as confirmed by the analysis of XRD pattern using the Scherrer equation. Then, the synthesized Cr_2_O_3_ was used as a heterogeneous Lewis acid catalyst for efficient synthesis of imidazole derivatives by condensation of aromatic aldehydes took place with ammonium acetate and benzil in the attendance of a catalytic amount of Cr_2_O_3_, and H_2_O as solvent under microwave illumination. High reaction yield (97%) was obtained when benzaldehyde was used as aldehyde derivative. The prepared nanocatalyst was recovered and its high efficiency even after six runs was proved. Reasonable reaction times, excellent yields, easy work-up, and the absence of any hazardous and volatile organic solvents were the main merits of this benign protocol.

## Supplementary Information

Supplementary Information.

## Data Availability

All data generated or analyzed during this study are included in this published article [and its supplementary information files].

## References

[1] Khan I, Saeed K, Khan I (2019). Nanoparticles: Properties, applications and toxicities. Arab. J. Chem..

[2] Ghotekar S, Pagar K, Pansambal S, Murthy HCA, Oza R (2020). A review on eco-friendly synthesis of BiVO_4_ nanoparticle and its eclectic applications. Adv. J. Sci. Eng..

[3] Ghotekar S, Pansambal S, Bilal M, Pingale SS, Oza R (2021). Environmentally friendly synthesis of Cr_2_O_3_ nanoparticles: Characterization, applications and future perspective—A review. Case Stud. Chem. Environ. Eng. (CSCEE).

[4] Abdullah MM, Rajab FM, Al-Abbas SM (2014). Structural and optical characterization of Cr_2_O_3_ nanostructures: Evaluation of its dielectric properties. AIP Adv..

[5] Hassan D, Khalil AT, Solangi AR, El-Mallul A, Shinwari ZK, Maaza M (2019). Physiochemical properties and novel biological applications of *Callistemon viminalis*-mediated α-Cr_2_O_3_ nanoparticles. Appl. Organomet. Chem..

[6] Ding C, Ma Y, Lai X, Yang Q, Xue P, Hu F, Geng W (2017). Ordered large-pore mesoporous Cr_2_O_3_ with ultrathin framework for formaldehyde sensing. ACS Appl. Mater. Interfaces.

[7] Wang S, Murata K, Hayakawa T, Hamakawa S, Suzuki F (2000). Dehydrogenation of ethane with carbon dioxide over supported chromium oxide catalysts. Appl. Catal. Gen..

[8] Ku RC, Winterbottom WL (1985). Electrical conductivity in sputter-deposited chromium oxide coatings. Thin Solid Films.

[9] Linder M, Hocker T, Holzer L, Friedrich KA, Iwanschitz B, Mai A, Schuler JA (2013). Cr_2_O_3_ scale growth rates on metallic interconnectors derived from 40,000 h solid oxide fuel cell stack operation. J. Power Sour..

[10] Sim JK, Lee SK, Kim JS, Jeong KU, Ahn HK, Lee CR (2016). Efficiency enhancement of CIGS compound solar cell fabricated using homomorphic thin Cr_2_O_3_ diffusion barrier formed on stainless steel substrate. Appl. Surf. Sci..

[11] Jianhua LI, Qingchi SUN (2008). Effects of Cr_2_O_3_ doping on the electrical properties and the temperature stabilities of PZT binary piezoelectric ceramics. Rare Met..

[12] Su J, Xue H, Gu M, Xia H, Pan F (2014). Synthesis of spherical Cr_2_O_3_ nanoparticles by a microwave refluxing method and their photocatalytic properties. Ceram. Int..

[13] Moezzi A, McDonagh AM, Cortie MB (2012). Zinc oxide particles: Synthesis, properties and applications. Chem. Eng. J..

[14] Pei Z, Xu H, Zhang Y (2009). Preparation of Cr_2_O_3_ nanoparticles via C_2_H_5_OH hydrothermal reduction. J. Alloys Compd..

[15] Li L, Zhu Z, Yao X, Lu G, Yan Z (2008). Synthesis and characterization of chromium oxide nanocrystals via solid thermal decomposition at low temperature. Micropor. Mesopor. Mat..

[16] Lima MD, Bonadimann R, Andrade MJ, Toniolo JC, Bergmann CP (2006). Evaluation of two different methods to synthesize cobalt-aluminate spinel. J. Eur. Ceram. Soc..

[17] Pinna N, Garnweitner G, Antonietti M, Niederberger M (2004). Non-aqueous synthesis of high-purity metal oxide nanopowders using an ether elimination process. Adv. Mater..

[18] Kim DW, Shin SI, Lee JD, Oh SG (2004). Preparation of chromia nanoparticles by precipitation–gelation reaction. Mater. Lett..

[19] Tsuzuki T, Mc Cormick PG (2000). Synthesis of Cr_2_O_3_ nanoparticles by mechanochemical processing. Acta Mater..

[20] Zhong ZC, Cheng RH, Bosley J, Dowben PA, Sellmyer DJ (2001). Fabrication of chromium oxide nanoparticles by laser-induced deposition from solution. Appl. Surf. Sci..

[21] Mougin J, Le Bihan T, Lucazeau G (2001). High-pressure study of Cr_2_O_3_ obtained by high-temperature oxidation by X-ray diffraction and Raman spectroscopy. J. Phys. Chem. Solids..

[22] Sone B, Manikandan E, Gurib-Fakim A, Maaza M (2016). Single-phase α-Cr_2_O_3_ nanoparticles’ green synthesis using *Callistemon viminalis*’ red flower extract. Green Chem. Lett. Rev..

[23] Cuong HN, Pansambal S, Ghotekar S, Oza R, Hai NTT, Viet NM, Nguyen V-H (2022). New frontiers in the plant extract mediated biosynthesis of copper oxide (CuO) nanoparticles and their potential applications: A review. Environ. Res..

[24] Marzban A, Mirzaei SZ, Karkhane M, Ghotekar SK, Danesh A (2022). Biogenesis of copper nanoparticles assisted with seaweed polysaccharide with antibacterial and antibiofilm properties against methicillin-resistant *Staphylococcus aureus*. J. Drug Deliv. Sci. Technol..

[25] Kashid Y, Ghotekar S, Bilal M, Pansamba S, Oza R, Varma RS, Nguyen V-H, Murthy HCA, Mane D (2022). Bio-inspired sustainable synthesis of silver chloride nanoparticles and their prominent applications. J. Indian Chem. Soc..

[26] Hassanisaadi M, Shahidi Bonjar AH, Rahdar A, Varma RS, Ajalli N, Pandey S (2022). Eco-friendly biosynthesis of silver nanoparticles using Aloysia citrodora leaf extract and evaluations of their bioactivities. Mater. Today Commun..

[27] Garibo D, Borbón-Nuñez HA, de León JND, García Mendoza E, Estrada I, Toledano-Magaña Y, Tiznado H, Ovalle-Marroquin M, Soto-Ramos AG, Blanco A, Rodríguez JA, Romo OA, Chávez-Almazán LA, Susarrey-Arce A (2020). Green synthesis of silver nanoparticles using *Lysiloma acapulcensis* exhibit high-antimicrobial activity. Sci. Rep..

[28] Sharma UR, Sharma N (2021). Green synthesis, anti-cancer and corrosion inhibition activity of Cr_2_O_3_ nanoparticles. Bioint. Res. App. Chem..

[29] Mohamed HEA, Afridi S, Khalil AT, Zohra T, Ali M, Alam MM, Ikram A, Shinwari ZK, Maaza M (2020). Phyto-fabricated Cr_2_O_3_ nanoparticle for multifunctional biomedical applications. Nanomedicine.

[30] Kotb OM, Abd El-Latif FM, Atawia AR, Saleh SS, El-Gioushy SF (2020). Green synthesis of chromium nanoparticles by aqueous extract of *Melia azedarach*, *Artemisia herba-alba* and bacteria fragments against *Erwinia amylovora*. Asian J. Biotech. Biores. Tech..

[31] Isacfranklin M, Ameen F, Ravi G, Yuvakkumar R, Hong SI, Velauthapillai D, Thambidurai M, Dang C (2020). Single-phase Cr_2_O_3_ nanoparticles for biomedical applications. Ceram. Int..

[32] Tsegay MG, Gebretinsae HG, Nuru ZY (2020). Structural and optical properties of green synthesized Cr_2_O_3_ nanoparticles. Mater Today Proc..

[33] Iqbal J, Abbasi BA, Munir A, Uddin S, Kanwal S, Mahmood T (2020). Facile green synthesis approach for the production of chromium oxide nanoparticles and their different in vitro biological activities. Microsc. Res. Tech..

[34] Sackey J, Morad R, Bashir AKH, Kotsedi L, Kaonga C, Maaza M (2021). Biosynthesised black α-Cr_2_O_3_ nanoparticles; experimental analysis and density function theory calculations. J. Alloys Compd..

[35] Ramesh C, Kumar KTM, Latha N, Ragunathan V (2012). Green synthesis of Cr_2_O_3_ nanoparticles using *Tridax procumbens* leaf extract and its antibacterial activity on *Escherichia coli*. Curr. Nanosci..

[36] Patra JK, Baek K-H (2014). Green nanobiotechnology: Factors affecting synthesis and characterization techniques. J. Nanomater..

[37] Pradeep M, Kruszka D, Kachlicki P, Mondal D, Franklin G (2022). Uncovering the phytochemical basis and the mechanism of plant extract-mediated eco-friendly synthesis of silver nanoparticles using ultra-performance liquid chromatography coupled with a photodiode array and high-resolution mass spectrometry. ACS Sustain. Chem. Eng..

[38] Singh PP, Jaiswal AK, Kumar A, Gupta V, Rakish B (2021). Untangling the multi-regime molecular mechanism of verbenol-chemotype Zingiber of cinale essential oil against *Aspergillus favus* and afatoxin B_1_. Sci. Rep..

[39] Gao X, Chen X, Zhang J, Guo W, Jin F, Yan N (2016). Transformation of Chitin and Waste Shrimp shells into acetic acid and pyrrole. ACS Sustain. Chem. Eng..

[40] Chen X, Chew SL, Kerton FM, Yan N (2014). Direct conversion of chitin into a *N*-containing furan derivative. Green Chem..

[41] Singh H, Rajput JK (2018). Co(II) anchored glutaraldehyde crosslinked magnetic chitosan nanoparticles (MCS) for synthesis of 2,4,5-trisubstituted and 1,2,4,5-tetrasubstituted imidazoles. Appl. Organomet. Chem..

[42] Marzouk AA, Abu-Dief AM, Abdelhamid AA (2018). Hydrothermal preparation and characterization of ZnFe_2_O_4_ magnetic nanoparticles as an efficient heterogeneous catalyst for the synthesis of multi-substituted imidazoles and study of their anti-inflammatory activity. Appl. Organomet. Chem..

[43] Takle AK, Brown MJB, Davies S, Dean DK, Francis G, Gaiba A, Hird AW, King FD, Lovell PJ, Naylor A, Reith AD, Steadman JG, Wilson DM (2006). The identification of potent and selective imidazole-based inhibitors of B-Raf kinase. Bioorg. Med. Chem. Lett..

[44] Balalaie S, Arabanian A, Hashtroudi MS (2000). Zeolite HY and Silica gel as new and efficient heterogenous catalysts for the synthesis of triarylimidazoles under microwave irradiation. Monatsh. Fur. Chem..

[45] Siddiqui SA, Narkhede UC, Palimkar SS, Daniel T, Lahoti RJ, Srinivasan KV (2005). Room temperature ionic liquid promoted improved and rapid synthesis of 2,4,5-triaryl imidazoles from aryl aldehydes and 1,2-diketones or α-hydroxyketone. Tetrahedron.

[46] Shaabani A, Rahmati A, Aghaaliakbari B, Safaei-Ghomi J (2006). 1,1,3,3-*N*,*N*,*N*′,*N*′-Tetramethylguanidinium trifluoroacetate ionic liquid–promoted efficient one-pot synthesis of trisubstituted imidazoles. Synth. Commun..

[47] Kidwai M, Mothsra P, Bansal V, Goyal R (2006). Efficient elemental iodine catalyzed one-pot synthesis of 2,4,5-triarylimidazoles. Monatsh. Fur. Chem..

[48] Sangshetti JN, Kokare ND, Kothakar SA, Shinde DB (2008). Sodium Bisulfite as an efficient and inexpensive catalyst for the one-pot synthesis of 2,4,5-triaryl-1*H*-imidazoles from benzil or benzoin and aromatic aldehydes. Monatsh. Fur. Chem..

[49] Sharma GVM, Jyothi Y, Lakshmi PS (2006). efficient room-temperature synthesis of tri- and tetrasubstituted imidazoles catalyzed by ZrCl_4_. Synth. Commun..

[50] Wang LM, Wang YH, Tian H, Yao YF, Shao H, Liu B (2006). Ytterbium triflate as an efficient catalyst for one-pot synthesis of substituted imidazoles through three-component condensation of benzil, aldehydes and ammonium acetate. J. Fluorine Chem..

[51] Balalaie S, Hashemi MM, Akhbari MA (2003). A novel one-pot synthesis of tetrasubstituted imidazoles under solvent-free conditions and microwave irradiation. Tetrahedron Lett..

[52] Shelke K, Kakade G, Shingate B, Shingare M (2008). Microwave induced one-pot synthesis of 2,4,5-tri aryl imidazoles using glyoxylic acid as a catalyst under solvent free condition. Rasayan J. Chem..

[53] Sharma SD, Hazarika P, Konwar D (2008). An efficient and one-pot synthesis of 2,4,5-trisubstituted and 1,2,4,5-tetrasubstituted imidazoles catalyzed by InCl_3_.3H_2_O. Tetrahedron Lett..

[54] Hangirgekar SP, Kumbhar VV, Shaikh AL, Bhairuba IA (2014). One-pot synthesis of 2,4,5-trisubstituted imidazoles using cupric chloride as a catalyst under solvent free conditions. Der Pharma Chemica..

[55] Kafi-Ahmadi L, Poursattar Marjani A, Nozad E (2021). Ultrasonic-assisted preparation of Co_3_O_4_ and Eu-doped Co_3_O_4_ nanocatalysts and their application for solvent-free synthesis of 2-amino-4*H*-benzochromenes under microwave irradiation. Appl. Organomet. Chem..

[56] Majidi Arlan F, Poursattar Marjani A, Javahershenas R, Khalafy J (2021). Recent developments in the synthesis of polysubstituted pyridines via multicomponent reactions using nanocatalysts. New. J. Chem..

[57] Azimi F, Poursattar Marjani A, Keshipour S (2021). Fe(II)-phthalocyanine supported on chitosan aerogel as a catalyst for oxidation of alcohols and alkyl arenes. Sci. Rep..

[58] Khashaei M, Kafi-Ahmadi L, Khademinia S, Poursattar Marjani A, Nozad E (2022). A facile hydrothermal synthesis of high-efficient NiO nanocatalyst for preparation of 3,4-dihydropyrimidin-2(1*H*)-ones. Sci. Rep..

[59] Parsa Habashi B, Poursattar Marjani A (2022). *N*-methylpyrrolidine as an effective organocatalyst for the regioselective synthesis of 3-hydroxy-3,5/6-di-aryl-1*H*-imidazo[1,2-*a*]imidazol-2(3*H*)-ones. Res. Chem. Intermed..

[60] Kafi-Ahmadi L, Khademinia S, Poursattar Marjani A, Gozali Balkanloo P (2022). Fabrication of 5-aryl-1*H*-tetrazoles derivatives by solid-state synthesized MgFe_2_O_4_ and MgFe_2_Zn_*x*_O_4+δ_ heterogeneous nanocatalysts. Res. Chem. Intermed..

[61] Chen L, Song Z, Wang X, Prikhodko SV, Hu J, Kodambaka S, Richards R (2009). Three-dimensional morphology control during wet chemical synthesis of porous chromium oxide spheres. ACS Appl. Mater. Interfaces..

[62] Farzaneh F, Najafi M (2011). Synthesis and characterization of Cr_2_O_3_ nanoparticles with triethanolamine in water under microwave irradiation. J. Sci. Islam. Repub. Iran..

[63] Chavan HV, Narale DK (2013). Synthesis of 2,4,5-triaryl and 1,2,4,5-tetraaryl imidazoles using silica chloride as an efficient and recyclable catalyst under solvent-free conditions. C. R. Chim..

[64] Chavan LD, Shankarwar SG (2015). KSF supported 10-molybdo-2-vanadophosphoric acid as an efficient and reusable catalyst for one-pot synthesis of 2,4,5-trisubstituted imidazole derivatives under solvent-free condition. Chin. J. Catal..

[65] Khazaei HA, Alavi Nik A, Ranjbaran AR, Moosavi-Zare, (2016). Synthesis, characterization and application of Ni_0.5_Zn_0.5_Fe_2_O_4_ nanoparticles for the one pot synthesis of triaryl-1*H*-imidazoles. RSC Adv..

[66] Nagalakshmi G (2008). Synthesis and pharmacological evaluation of 2-(4-halosubstituted phenyl)-4,5-diphenyl-1*H*-imidazoles. Eur. J. Chem..

[67] Gorsd M, Sathicq G, Romanelli G, Pizzio L, Blanco M (2016). Tungstophosphoric acid supported on core-shell polystyrene-silica microspheres or hollow silica spheres catalyzed trisubstituted imidazole synthesis by multicomponent reaction. J. Mol. Catal. A. Chem..

[68] Chundawat TS, Sharma N, Kumari P, Bhagat S (2016). Microwave-assisted nickel-catalyzed one-pot synthesis of polysubstituted imidazoles. Synlett.

[69] Thimmaraju N, Shamshuddin SZM (2016). Synthesis of 2,4,5-trisubstituted imidazoles, quinoxalines and 1,5-benzodiazepines over eco-friendly and highly efficient ZrO_2_-Al_2_O_3_ catalyst. RSC Adv..

[70] Shitole BV, Shitole NV, Ade SB, Kakde GK (2015). Microwave-induced one-pot synthesis of polysubstituted imidazoles using Rochelle salt as a green novel catalyst. Orbital. E- J. Chem..

[71] Mardani HR, Forouzani M, Emami R (2019). Efficient and green synthesis of trisubstituted imidazoles by magnetically nanocatalyst and microwave assisted. Asian J. Green Chem..

[72] Safari J, Dehghan Khalili S, Banitab SH (2011). Three-component, one-pot synthesis of polysubstituted imidazoles catalyzed by TiCl_4_–SiO_2_ under conventional heating conditions or microwave irradiation. Synth. Commun..

[73] Asressu KH, Chan C-K, Wang C-C (2021). TMSOTf-catalyzed synthesis of trisubstitutedimidazoles using hexamethyldisilazane as a nitrogen source under neat and microwave irradiation conditions. RSC Adv..

[74] Alikarami M, Amozad M (2017). One-pot synthesis of polysubstituted imidazole derivatives catalyzed by btppc under solvent-free conditions. Bull. Chem. Soc. Ethiop..

[75] Safari J, Gandomi-Ravandi S, Akbari Z (2013). Improving methodology for the preparation of highly substituted imidazoles using nano-MgAl_2_O_4_ as catalyst under microwave irradiation. Iran. J. Catal..

