# Supplementary material for: Microwave-assisted preparation of polysubstituted imidazoles using Zingiber extract synthesized green Cr2O3 nanoparticles
Source: Sci Rep. 2022 Nov 19;12:19942. doi: 10.1038/s41598-022-24364-6 (PMC9675835; doi:10.1038/s41598-022-24364-6)
Supplement: Supplementary file 1 — Supplementary Information. [file 41598_2022_24364_MOESM1_ESM.pdf]

Supporting Information

Microwave-assisted preparation of polysubstituted imidazoles using Zingiber extract synthesized green Cr_2O_3 nanoparticles

Leila Kafi-Ahmadi[1], Shahin Khademinia[2], Ahmad Poursattar Marjani[3,*] & Ehsan Nozad[3]

[1]*Department of Inorganic Chemistry, Faculty of Chemistry, Urmia University, Urmia, Iran.*

[2]*Department of Inorganic Chemistry, Faculty of Chemistry, Semnan University, Semnan, Iran.*

[3]*Department of Organic Chemistry, Faculty of Chemistry, Urmia University, Urmia, Iran.*

**E-mail: a.poursattar@urmia.ac.ir; a.poursattar@gmail.com*

2,4,5-Triphenyl-1*H*-imidazole (Table 5, entry 1)

White solid; FT-IR (KBr, *v*, cm^{-1}): 3446, 3084, 1596, 1620, 1604, 1491, 1465, 1189, 1120, 950, 761, 732, 506; ^{1}H-NMR (400 MHz, DMSO-d_6) δ (ppm): 7.25–7.64 (m, 13H, ArH), 8.13–8.16 (m, 2H, ArH), 12.65 (s, 1H, NH). Anal. Calcd. for $C_{21}H_{16}N_2$ (%): C (85.13), H (5.40), N (9.45), Found: C (85.00), H (5.13), N (9.26).

2-(4-Bromophenyl)-4,5-diphenyl-1*H*-imidazole (Table 5, entry 2)

White solid; FT-IR (KBr, *v*, cm^{-1}): 3439, 3082, 1593, 1620, 1605, 1486, 1462, 1180, 1113, 941, 762, 730, 503; ^{1}H-NMR (400 MHz, DMSO-d_6) δ (ppm): 7.40–7.70 (m, 12H, ArH), 8.03–8.15 (m, 2H, ArH), 12.73 (s, 1H, NH). Anal. Calcd. for $C_{21}H_{15}BrN_2$ (%): C (67.21), H (4.03), N (7.47). Found: C (67.11), H (3.95), N (7.26).

2-(2-Chlorophenyl)-4,5-diphenyl-1*H*-imidazole (Table 5, entry 3)

White solid; FT-IR (KBr, *v*, cm^{-1}): 3438, 3089, 1587, 1619, 1600, 1488, 1465, 1182, 1114, 946, 760, 732, 501; ^{1}H-NMR (400 MHz, DMSO-d_6): δ (ppm): 7.30 (t, *J* = 6.9 Hz, 1H, ArH), 7.32–7.39 (m, 3H, ArH), 7.42–7.56 (m, 6H, ArH), 7.58 (d, *J* = 7.6 Hz, 2H, ArH), 7.60–7.64 (m, 1H, ArH), 7.81–7.82 (m, 1H, ArH), 12.59 (bs, NH, 1H). Anal. Calcd. for $C_{21}H_{15}ClN_2$ (%): C (76.26), H (4.53), N (8.47), Found: C (76.14), H (4.22), N (8.24).

2-(4-Chlorophenyl)-4,5-diphenyl-1*H*-imidazole (Table 5, entry 4)

White solid; FT-IR (KBr, *v*, cm^{-1}): 3441, 3083, 1590, 1621, 1603, 1491, 1467, 1188, 1116, 950, 762, 735, 502; ^{1}H-NMR (400 MHz, DMSO-d_6) δ (ppm): 7.24–7.54 (m, 12H, ArH), 8.10–8.16 (m, 2H, ArH), 12.72 (s, 1H, NH). Anal. Calcd. for $C_{21}H_{15}ClN_2$ (%): C (76.26), H (4.53), N (8.47), Found: C (76.01), H (4.17), N (8.19).

2-(4-Fluorophenyl)-4,5-diphenyl-1*H*-imidazole (Table 5, entry 5)

White solid; FT-IR (KBr, *v*, cm^{-1}): 3439, 3087, 1589, 1620, 1604, 1489, 1468, 1180, 1111, 940, 761, 733, 502; ^{1}H-NMR (400 MHz, DMSO-d_6) δ (ppm): 7.26–8.14 (m, 12H, ArH), 8.10–8.15 (m, 2H, ArH), 12.54 (s, 1H, NH). Anal. Calcd. for $C_{21}H_{15}FN_2$ (%): C (80.25), H (4.77), N (8.91). Found: C (80.09), H (4.40), N (8.76).

2-(4-Hydroxyphenyl)-4,5-diphenyl-1*H*-imidazole (Table 5, entry 6)

White solid; FT-IR (KBr, *v*, cm^{-1}): 3440, 3081, 1592, 1621,1602, 1490, 1466, 1187, 1119, 951, 763, 734, 504; ^{1}H-NMR (400 MHz, DMSO-d_6) δ (ppm): 6.90–8.33 (m, 12H, ArH), 8.20–8.33 (m, 2H, ArH), 9.64 (s, 1H, OH), 12.32 (s, 1H, NH). Anal. Calcd. for $C_{21}H_{16}N_2$ (%): C (80.76), H (5.12), N (8.97), Found: C (80.29), H (5.03), N (8.66).

4,5-Diphenyl-2-*p*-tolyl-1*H*-imidazole (Table 5, entry 7)

White solid; FT-IR (KBr, *v*, cm^{-1}): 3441, 3081, 1585, 1623, 1606, 1483, 1466, 1185, 1117, 944, 760, 730, 501; ^{1}H-NMR (400 MHz, DMSO-d_6) δ (ppm): 2.37 (s, 3H, CH_3), 7.23–7.62 (m, 12H, ArH), 8.00–8.06 (m, 2H, ArH), 12.66 (s, 1H, NH). Anal. Calcd. for $C_{22}H_{18}N_2$ (%): C (85.16), H (5.80), N (9.03). Found: C (84.89), H (5.31), N (8.86).

2-(2-Methoxyphenyl)-4,5-diphenyl-1*H*-imidazole (Table 5, entry 8)

White solid; FT-IR (KBr, *v*, cm^{-1}): 3440, 3087, 1589, 1623, 1608, 1483, 1455, 1178, 1122, 956, 755, 734, 507; ^{1}H-NMR (400 MHz, DMSO-d_6) δ (ppm): 3.90 (s, 3H, CH_3), 7.06 (t, *J* = 7.5 Hz, 1H, ArH), 7.16–7.28 (m, 2H, ArH), 7.30 (t, *J* = 6.9 Hz, 2H, ArH), 7.40–7.49 (m, 6H, ArH), 7.54 (d, *J* = 7.6 Hz, 2H, ArH), 8.15 (d, *J* = 7.2 Hz, 1H, ArH), 11.87 (bs, 1H, NH). Anal. Calcd. for $C_{22}H_{18}N_2O$ (%): C (80.96), H (5.56), N (8.58). Found: C (80.49), H (5.15), N (8.41).

2-(4-Methoxyphenyl)-4,5-diphenyl-1*H*-imidazole (Table 5, entry 9)

White solid; FT-IR (KBr, *v*, cm^{-1}): 3445, 3083, 1586, 1625, 1602, 1480, 1460, 1181, 1120, 950, 758, 732, 500; ^{1}H-NMR (400 MHz, DMSO-d_6) δ (ppm): 3.65 (s, 3H, CH_3), 7.05–7.57 (m, 12H, ArH), 8.05–8.16 (m, 2H, ArH), 12.72 (bs, 1H, NH). Anal. Calcd. for $C_{22}H_{18}N_2O$ (%): C (80.96), H (5.56), N (8.58), Found: C (80.56), H (5.24), N (8.38).

2-(3-Nitrophenyl)-4,5-diphenyl-1*H*-imidazole (Table 5, entry 10)

Pale yellow solid; FT-IR (KBr, *v*, cm^{-1}): 3440, 3085, 1590, 1619, 1600, 1485, 1464, 1182, 1112, 942, 763, 731, 504; ^{1}H-NMR (400 MHz, DMSO-d_6) δ (ppm): 7.36–8.54 (m, 13H, ArH), 8.88 (s, 1H, ArH), 13.07 (s, 1H, NH). Anal. Calcd. for $C_{21}H_{15}O_2N_3$ (%): C (73.90), H (4.39), N (12.31). Found: C (72.98), H (4.07), N (11.97).
